# Phenotypic and molecular basis for genetic variation in jelly palms (*Butia* sp.): where are we now and where are we headed to?

**DOI:** 10.1590/1678-4685-GMB-2023-0145

**Published:** 2023-11-10

**Authors:** Camila Fritzen Cidón, Andreia Carina Turchetto-Zolet, Miklos Maximiliano Bajay, Maria Imaculada Zucchi, Enéas Ricardo Konzen

**Affiliations:** 1 Universidade Federal do Rio Grande do Sul, Programa de Pós-Graduação em Genética e Biologia Molecular, Porto Alegre, RS, Brazil.; 2 Universidade Federal do Rio Grande do Sul, Departamento de Genética, Porto Alegre RS, Brazil.; 3 Universidade do Estado de Santa Catarina, Centro de Educação Superior da Região Sul, Laguna, SC, Brazil.; 4 Agência Paulista de Tecnologia dos Agronegócios, Laboratório de Conservação Genética e Genômica, Piracicaba, SP, Brazil.; 5 Universidade Federal do Rio Grande do Sul, Departamento Interdisciplinar, Centro de Estudos Costeiros, Limnológicos e Marinhos, Campus Litoral Norte, Imbé, RS, Brazil.

**Keywords:** Genetic diversity, phenotypic variation, endangered species, palm trees, conservation genetics

## Abstract

We compiled studies that addressed morphological and physicochemical traits, as well as population genetic studies involving jelly palms, genus *Butia* (Arecaceae). First, we conducted a bibliometric study with selected articles, by revising the fundamental contributions to unraveling phenotypic traits that have been used for describing the phenotypic variation within and among populations. Moreover, we sought to comprehend the patterns of genetic diversity and structure that have been presented so far, based on molecular markers. Finally, we conducted a review of the gene sequences registered to NCBI for *Butia*. Overall, morphological descriptors have been proposed to depict population-level variability, but the most significant results are available from chemical properties and characterization of metabolites, revealing important traits to being explored. Yet, limited information is available to describe population variation and their genetic components. On the molecular level, almost all studies so far provided results with classical molecular markers. The literature of SNP markers for *Butia* species is virtually non-existent. Given the current endangered state of *Butia* species, it is urgent that researchers pursue updated genomic technologies to invest in in-depth characterizations of the genetic diversity and structure of jelly palms. The current state of population fragmentation urges effective measures toward their conservation.

## Introduction

*Butia* (Becc.) Becc. is a genus of palm trees (Arecaceae) autochthonous to South America. Trees are popularly known as “butiás”, pindo palms or jelly palms. The species are naturally distributed in southern and central Brazil, eastern Paraguay, northeastern Argentina and northwestern and southeastern Uruguay (Lorenzi et al., 2010). Their occurrence in Brazil encompasses a few populations at the southeast of Bahia, east of Goiás and northern Minas Gerais, but most populations are found in the southern states, especially in Rio Grande do Sul and the south of Santa Catarina (Marcato, 2004; Soares and Longhi, 2011; Müller et al., 2012; Soares *et al.*, 2014; dos Santos et al., 2017). *Butia* species can also be found in São Paulo, Mato Grosso do Sul and Paraná (Reitz, 1974; Eslabão et al., 2016; Barbieri et al., 2017). Their occurrence encompasses the Pampa, Atlantic Forest and Cerrado biomes (Hoffmann et al., 2017).

Jelly palms can reach 10 meters or more in height, with seasonal reproduction, characterized by monoecious, protandrous and allogamous plants, rarely showing geitonogamy (Mercadante-Simões et al., 2006; Corrêa et al., 2009; Beskow et al., 2015; Sosinski et al., 2019). The fruit clusters are composed of dozens or even more than a thousand fruits. Fruits vary in shape from ovoid to globose, fibrous, sweet, slightly acidic and are rich in phenolic compounds, carotenoids, vitamin C and potassium. Fruits turn yellow, orange or reddish when mature (Lorenzi et al., 2010; Beskow *et al.*, 2015) and are quite versatile in usage by local communities, being employed in the manufacture of juices, liqueurs, ice cream, and jellies (Hoffmann et al., 2014; dos Santos et al., 2017). The pulp is composed of the exocarp and mesocarp, containing high concentrations of carbohydrates, fiber, pro vitamin A, vitamin C, carotenoids, and phenolic compounds, which offer potential for agroindustry expansion in the utilization of the fruit (Faria et al., 2008a; Pereira et al., 2013).

The number of species counted within the genus *Butia* has changed considerably over frequent taxonomic debates based on morphological traits. The work by Noblick (2010) acknowledged 18 species, among them *Butia capitata* (Mart.) Becc., that was first identified in 1826 as *Cocos capitata*. Originally, Martius described the palm from their observations near the town of Montes Claros, in Minas Gerais state (Noblick, 2011). In general, this palm occurs in Cerrado areas, typically in sandy soils in Bahia, Goiás and Minas Gerais states (Noblick, 2010). A few authors, however, also attributed the name *Butia capitata* to a set of populations that occur in the coastal plain of Uruguay, Rio Grande do Sul and Santa Catarina states, in Brazil (Rosa et al., 1998; Soares et al., 2014). However, a recent reclassification was described for the populations of Uruguay and southern Brazil, and those located in Cerrado. *B. capitata* was then attributed only to the populations from Cerrado and *B. odorata* became the designated species for populations to the south of a small municipality called Osório, in Rio Grande do Sul state (approximately at latitude of 29^o^S). All populations north from that and located by the coastal plain of the Atlantic are designated as *B. catarinensis* (Noblick, 2010; Noblick, 2011). Another study recognized *B. poni* as a novel species (Deble et al., 2017) and, more recently, a new endemic and endangered species has been described in a narrow area from Brazilian Cerrado, *B. buenopolensis* (Noblick and Sant’anna-Santos, 2021). 

The taxonomic delimitation of *Butia* species based on morphological diversity is a difficult task, given the full evolutionary activity of the species, which are still in the process of establishing morphological characters and the high degree of genetic variation in morphological, phenological and physicochemical traits (Soares 2013; Soares *et al.*, 2014; Beskow et al., 2015).

In general, the species are threatened, and some are already at risk of extinction due to the fragmentation of their habitat by expansion of urban areas, agricultural activities that replace natural palm trees, removal and illegal commercialization of plants, reforestation with other tree species, and limited natural regeneration due to cattle grazing (Nazareno and dos Reis, 2014; Eslabão et al., 2016). This may have severe consequences, as habitat loss and fragmentation may reduce gene flow and genetic diversity, leading to inbreeding depression and reduced reproductive fitness (Reed and Frankham, 2003; Browne and Karubian, 2018). 

Genetic diversity is one of the three components of biodiversity recognized by the World Conservation Union (IUCN) as worthy of conservation. The need to conserve genetic diversity within populations is based on two premises: (i) genetic diversity is a key factor for evolution to occur; (ii) and the expected relationship between heterozygosity and population fitness, that is, genetic variation an important factor for fitness (Reed and Frankham, 2003). Molecular genetic characterization is essential for the delineation of effective conservation measures, that is, the rational use of germplasm resources and conservation management of endangered species (Mable, 2019; Cao et al., 2022; Aswathi et al., 2023). To date, population genetic studies on jelly palms based on molecular markers are yet scarcely available. In this review, a compilation of the studies shows articles with traditional molecular markers such as RAPD (random amplified polymorphic DNA), AFLP (amplified fragment length polymorphism), ISSR (inter-simple sequence repeats) and SSR (simple sequence repeats). 

Equally important for conservation, management and breeding of jelly palms is a deeper knowledge on phenological patterns as well as the reproductive biology of populations (Nazareno and dos Reis, 2014; Guilherme et al., 2015). Morphometric characterization of fruits and seeds is important for taxonomic studies, allowing the identification of varieties with economic value, verification of the phenotypic and genetic variation occurrence and association with the environment, both within and between plant populations (Padilha et al., 2016; Rios et al., 2016). Fruit and seed size variability within and among populations are an important component for studying the adaptation and evolution of trees in tropical and subtropical ecosystems (Candido-Ribeiro et al., 2019). For *Butia* palms, a wide phenotypic diversity is observed, which provides fruits varying for biometric, physicochemical and sensorial traits (Dal Magro *et al.*, 2006, Sganzerla, 2010). 

This review was developed to address the current state of art of knowledge on phenotypic and genetic variation on *Butia* species, considering three fundamental categories: (i) plant morphology, (ii) physicochemical properties and nutrition, and (iii) population genetic studies and cytogenetics. Moreover, we searched the NCBI database to look into gene sequences that involved *Butia* palms. Our main goal is to provide a panorama of the fundamental knowledge acquired through the years and prospect their utility for novel endeavors toward conservation and breeding of jelly palms.

## Material and Methods

### Sistematic review

A bibliometric study and a systematic review of articles were employed to encompass the knowledge produced about the morphological, physicochemical and molecular variation of jelly palm species. Four main steps were conducted in our survey: (i) keyword search through bibliometric platform; (ii) article selection after analyzing title and abstract, avoiding irrelevant contents to our subjects as well as redundant publications; (iii) categorization of papers according to scope. For that, three main categories were pre-defined: plant morphology, physicochemical properties and nutrition and population genetic and cytogenetic studies; (iv) categorization according to the object of research, journal of publication, year, objectives, main results and species involved.

The review was conducted through the “Web of Science” data platform, using the keyword “Butia” to search for all the results published up to 2023. The choice of “Web of Science” was due to the feasibility of conducting the search on the platform and the full access to the published articles. Our search presented 227 results, which were analyzed for title, abstract, and keywords. After reading and manual filtering, 47 articles were excluded for not being related to the *Butia* genus (e.g. papers that mentioned a municipality that contains the word “Butia”; or the word was found in the text but from other contexts not related to our goals). One article was excluded for not being available on the platform and another was excluded for being written in German. 

After this selection, 178 papers were analyzed concerning the three different categories here proposed:

1. Plant morphology: papers with phenology of the genus, biometric variables or anatomical and, eventually, even some physiological studies;
2. Physicochemical properties and nutrition: papers addressing chemical compounds in fruits, nutritional analyses and chemical composition of fruits and seeds;
3. Population genetics and cytogenetics: karyotype description, genetic diversity and structure.

After classification, 68 papers were selected. Of the total, 13 articles featured morphological analyses, 40 specifically dealt with physicochemical properties and nutrition, 11 referred to population genetic or cytogenetic studies, and 4 could be fitted into more than one category (e.g. morphological and physicochemical properties). 

Following bibliometric approaches, further analyses of the articles were conducted to determine: (i) a list of publication sources and the distribution of articles among them; (ii) discrimination of journal impact factors; (iii) production per year from the first to last publication date; (iv) ratios of publications per period and the categories defined as per our study; (v) ratio of annual publications per number of citations of each article. 

The qualitative analysis of the studies sought the validation and interpretation of scientific evidence relevant to the topics studied, and the identification of gaps in the literature, which may guide future studies. A few additional studies were also considered for the purpose of contextualization or discussion. 

### NCBI/Genbank search for sequences

We also searched the GenBank from NCBI (https://www.ncbi.nlm.nih.gov/popset/?term=butia) in order to find all the sequence information already available. Searching for the term “Butia”, we found 54 results (on April 26, 2023) in population sets (PopSets). After manually filtering the results, excluding non-related subjects, 43 popsets were checked for the gene sequences and the species involved.

### Resources available from GBIF and iNaturalist

Public databases on records for *Butia* species were also searched from GBIF (https://www.gbif.org/species/2736210) and iNaturalist (https://www.inaturalist.org/observations?place_id=any&taxon_id=180225) as of August 3^rd^, 2023. 

## Results

### Bibliometric study and public records on databases

Among the 68 articles that matched the scope of this research, 39 papers were published each in different journals (Table S1). The remaining 29 articles are distributed among 10 journals: “Revista Brasileira de Fruticultura” (Number of papers = 8), “Food Research International” (N = 4), “Food Bioscience” (N = 3), “Food Analytical Methods” (N = 2), “Ciência Rural” (N = 2), “American Journal of Botany” (N = 2), “Biota Neotropica” (N = 2), “Journal of Heredity” (N = 2), “Brazilian Journal of Biology” (N = 2) and “Food Science and Technology” (N = 2). Table S1 shows the list with all journals, number of publications and corresponding impact factor. 

The highest impact factor (JCR 2021 list; as on April 27^th^, 2023) among all journals was for “Energy Conversion and Management” (IF = 11.5) and the lowest was for “Revista Chilena de Nutricion” (IF = 0.1). The highest number of studies in frequency (N = 8) were published in a low impact journal (IF = 1.1) - “Revista Brasileira de Fruticultura”. Other four publications (N = 4) are available from a journal with a high impact factor (IF = 7.4) - “Food Research International”. 

**Figure 1 -:**
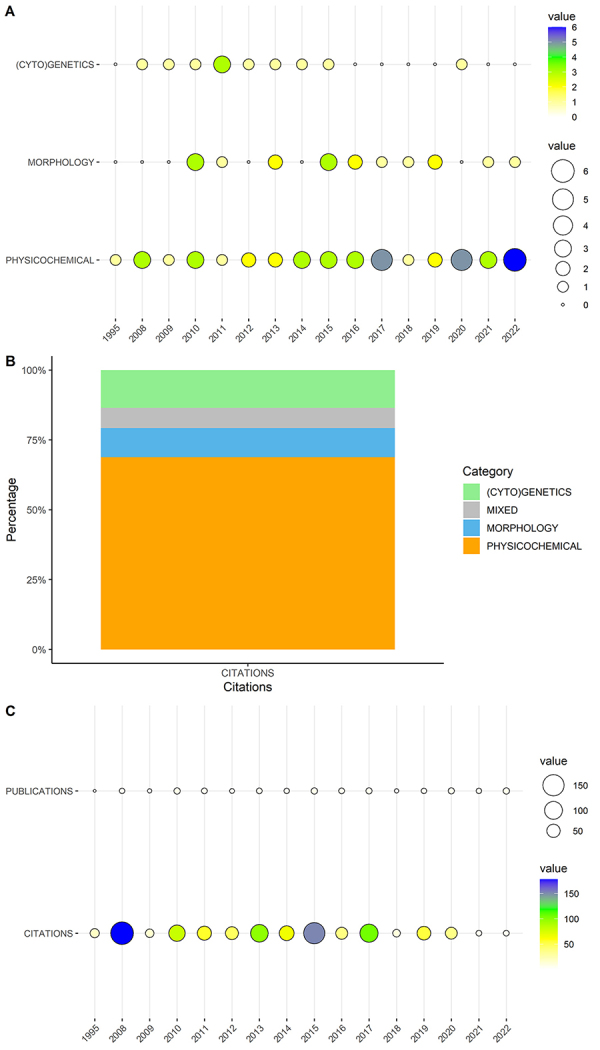
Bibliometric analyses of papers related to *Butia* palm species available from Web of Science. a - Number of papers published per year that involved jelly palms, based on our categorization in three main types of articles: (i) population genetics and cytogenetics; (ii) plant morphology; and (iii) physicochemical properties and nutrition. b - Proportion of the total number of articles per category. c - Number of publications and citations in all years that had at least one publication since 1995.

The average number of published papers per year was 2.42. Most of the articles (N = 40) were published in the last eight years, with the highest number (N = 7) in 2022, and 25% were published in the last three years. Figure 1a presents the distribution of publications per year according to the category of the paper. From the date of the first study (1995) to the second (2008), there were 13 years without any publication regarding morphology, physicochemical properties and nutrition or population genetics and cytogenetics. In the period from 2008 to 2022, publications were available over all consecutive years, with an average of 4 publications per year. From 1995 to 2022, articles dealing with jelly palm physicochemical properties and nutrition have been published in all years in that publications were available and retrieved from our survey (Figure 1a). 

As per our categorization, most studies in the period were devoted to physicochemical and nutritional analyses (N = 39). Plant morphology was the object of 18 studies, while population genetics and cytogenetics involved 12 articles. Among all papers, four were placed into more than one category, as they dealt with both morphology and physicochemical properties of jelly palms. Based on the frequency per category, an increase in physicochemical and nutritional studies is notable for the last five years (N = 17). 

The selected articles were cited 971 times throughout the entire period (updated up to April 2023), with the highest number of citations corresponding to studies of physicochemical properties and nutrition (N = 670), followed by population genetics and cytogenetics (N = 132) and plant morphology (N = 98) (Figure 1b). The articles that fitted into more than one category were classified as “Mixed” and cited 71 times in total. Figure 1c demonstrates the total number of citations and publications per year, considering all papers selected for this study. The highest number of published papers occurred in 2010, 2015 and 2022 (N = 7). The year 2008 received the highest number of citations (178 citations). The most cited paper was of Genovese et al. (2008) (125 citations), that characterized bioactive compounds contents and antioxidant capacity of Brazilian exotic fruits, one of them being *B. capitata*. The second most cited article (103 citations) was of Denardin et al. (2015), which also evaluated bioactive compounds and antioxidant properties for *B. eriospatha*. In total, 8 articles received no citations until the time of this research and have been published in journals with an impact factor from 0.9 to 7.4. 

**Figure 2 -:**
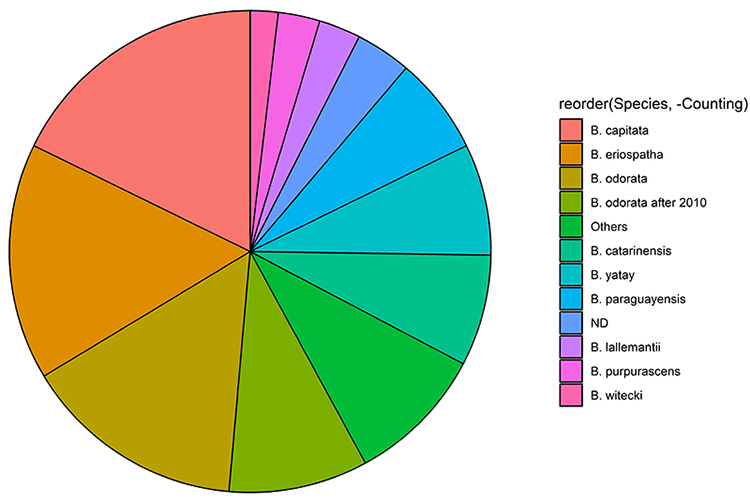
Relative contribution of jelly palm species encompassed in our review. As per a change in taxonomical identification, 10 papers were indicated separately as they ID the studied species as *B. capitata*, but currently they should be identified as *B. odorata*.

As for the jelly palm species studied, Figure 2 shows that most of them (N = 29) were about *B. capitata*, followed by *B. eriospatha* (N = 17) and *B. odorata* (N = 16). In fact, the number of papers that actually studied *B. odorata* was higher. This is because before 2010, *B. capitata* encompassed both *B. capitata* and *B. odorata*. It was Noblick (2010) that redefined the scientific names, considering populations of Rio Grande do Sul and Uruguay as *B. odorata*, while those located in Cerrado and areas from Central Brazil remained as *B. capitata*. That taken into consideration, we counted the number of papers that studied *B. odorata* to 26 and that studied *B. capitata* to 19, as there were 10 articles which named *B. odorata* as *B. capitata* of samples from Rio Grande do Sul state. However, that may depend on the type of paper, especially for those that were more concerned with physicochemical analyses rather than the specifics of the sample origin. Other papers were dedicated to study *B. catarinensis* (N = 8), *B. yatay* (N = 8), *B. paraguayensis* (N = 7), *B. lallemantii* (N = 3), *B. purpurascens* (N = 3) and *B. witecki* (N = 2). *B. archeri, B. campicola, B. exospadix, B. leiospatha, B. leptospatha, B. lepidotispatha, B. marmorii, B. matogrossensis, B. microspadix* and *B. pubispatha* were involved in only one publication (Sant’anna-Santos et al., 2018).

Three studies are review articles, so they did not focus on a species to attend the classification, as well as one study by Paroul et al. (2009), about wax hydrocarbon fractions. The authors did not specify the species but only the location, which does not ensure to state which species was investigated. The description of the main results and defined category of the 68 selected articles were summarized in Table S2. 

Besides research articles, we also checked GBIF and iNaturalist databases for occurrences that were registered. By searching “Butia” on GBIF, 2,731 occurences were found as of August 3^rd^ 2023 for 27 species. Of the total, 1,437 records were georeferenced and 924 occurences are accompanied by photographs. For instance, the same search was conducted with iNaturalist, resulting in 853 observations for 14 species, frequently accompanied by photograps, at the same date of search that GBIF. 

### Phenotypic traits based on plant morphology

Plant morphological traits have been the object of several studies conducted with jelly palms. From our interpretation, the studies were frequently devoted to describing differences among populations, sometimes leading to the description of novel species. On the other hand, work had been done to unravel phenotypic differences for several traits in studies aimed at conservation and breeding strategies for jelly palms. For that purpose, morphological traits have frequently been compared among preserved and managed populations at distinct environments. 

**Figure 3 -:**
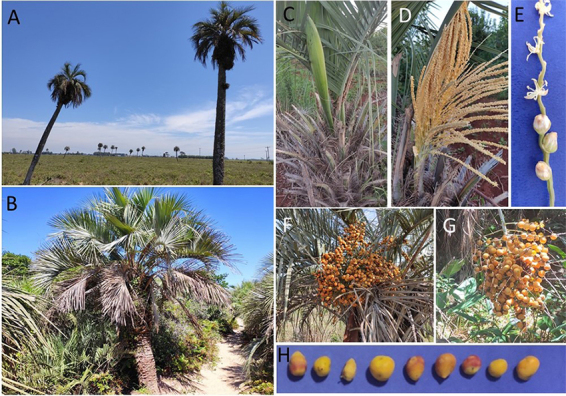
Basic characteristics of *Butia odorata* and *B. catarinensis* a - *B. odorata* individuals *in situ.* b - *B. catarinensis* individual *in situ.* c - Spathe of an *B. odorata* individual. d - General aspect of an *B. odorata* inflorescence. e- Detail of opened male flowers (upper) and female flowers (bottom) in a raquile. f- Fruit bunch of *B. odorata*. g- Fruit bunch of *B. catarinensis*. h- Sample of size and color variation of *B. catarinensis* fruits.

The distinction among species of *Butia* is frequently based on only a few morphological characteristics, such as between *B. catarinensis* and *B. odorata* (Figure 3). *B. odorata* can exceed 10m in adult individuals (Figure 3a). *B. catarinensis* individuals often do not exceed 2m in height (Figure 3b). Their distinction is also based on the spathe shape (Figure 3c). Although both species are monoecious, with male flowers more numerous than female flowers (Figure 3d,e), fruits o *B. odorata* are often round or slightly ovoid (Figure 3f), while *B. catarinensis* fruits are usually smaller and more elongated (Figure 3g, h) (Soares et al., 2014).

At the ultrastructural level, Sant’anna-Santos et al. (2015) compared the laminar anatomy between *B. capitata* and *B. odorata* and found some differences that they deemed useful for the distinction of the species. They observed that raphides were only found in*B. capitata* as well as small bundles of the midrib that fully surrounded the fibrous cylinder only in*B. odorata*. 

In a posterior study, the leaf anatomy of 18 species of *Butia* were evaluated. *B. marmorii* and *B. matogrossensis* showed unique characters: *B. marmorii* presented peculiar leaf anatomy represented by three exclusive anatomical characters, while discontinuity points within the fibrous ring of the midrib were exclusive to *B. matogrossensis*. The presence of raphides in*B. catarinensis*were deemed useful to distinguish it from *B. odorata* and *B. eriospatha* (Sant’anna-Santos et al., 2018). Pollen morphology and viability was studied in only one paper, which analyzed *B. odorata, B. eriospatha, B. yatay* and *B. paraguayensis*. Mourelle et al. (2016) concluded that all species produced monosulcate pollen grains with perforated exine, and pollen viability among species was shown not to be a limiting factor for the continuity of the species. 

A new species called *B. witeckii*, from the central of Rio Grande do Sul state, was identified only by the analysis of biometric and anatomical differences in comparison with the species *B. yatay* and *B. paraguayensis* (Soares and Longhi, 2011). In the study of the leaf anatomy of the species *B. paraguayensis, B. eriospatha, B. yatay* and *B. odorata*, Noblick and Sant’anna-Santos (2021) observed considerable variation in the characteristics evaluated among the species and suggested the revision of characters that have been used in the taxonomical keys used for species differentiation. 

Morphological traits have also been used as diagnostic tools for conservation. As local human populations use jelly palms leaves for making brooms in Brazilian Cerrado, leaf extraction may damage the structure and dynamics of jelly palms populations. In fact, leaf harvesting sites produced significantly fewer leaves, spurs, inflorescences and infructescence than preserved sites (Guilherme et al., 2015). In fragmented populations of *B. purpurascens*, morphometric differences among populations were notorious for fruit length, width, and weight. The authors suggested the possibility of deleterious genetic effects by interrupting gene flow between populations in fragmented ecosystems (Rocha et al., 2022).

By phenotyping morphological characteristics of distinct *Butia* species, studies have revealed the influence of environments and genotypes on fruit clusters composition and their productivity. After comparing fruit productivity between forest and grassland habitats for *B. eriospatha* populations, lower average production of infructescence and lower proportion of pulp per fruit was identified in the forest environment. The transformation of one population located in an Araucaria Forest to an open grassland environment may have shifted phenotypic variation related to fruit morphology and reproduction. Changes in fruit production, seedling survival and seed dispersal could have occurred, affecting the dynamics of the local population (Candido-Ribeiro et al., 2019). In a population of *B. capitata* in the Cerrado biome, fruit productivity differed among the sites evaluated and was directly related to the weight of the individuals and their leaf mass (da Silva and Scariot, 2013). In evaluating three different populations of *B. capitata* in Santa Vitória do Palmar - Rio Grande do Sul, researchers also found variations in fruit morphology, ratio of total soluble solids to total acidity and yield (Schwartz et al., 2010). *B. odorata* also differed concerning morphological and physicochemical aspects between populations from Santa Rosa and Santa Maria, southern Brazil (Ferrão et al., 2013). The nutritional characteristics of soils also appear to have direct implications in fruit productivity. In a study by Schlindwein et al. (2017) with *B. odorata*, more fertile soils were associated with higher productivity.

In the evaluation of 11 genotypes of *B. capitata*, Nunes et al. (2010) were successful in differentiating in relation to size, weight, number of fruits, firmness, color, acidity, and total soluble solids. Different characteristics in *B. capitata* were also recognized for follicles, nuts, drupes, berries, capsules and pyrens (Bobrov and Romanov, 2019). In a study with *B. odorata*, simultaneous analyses of yield and bioactive compounds were performed, however the most productive genotype did not coincide with the richest in bioactive compounds. Thus, fruit yield and bioactive phytochemical content appeared to be inversely proportional (Beskow et al., 2015). 

In *B. odorata* individuals preserved *in situ*, different morphological characters were identified based on leaf arrangement on the plant, stem circumference, leaf color, rachilla color, number of clusters, ripe fruit color, fruit shape, presence of fiber in the pulp, fruit diameter, flowering and fruiting time (Mistura et al., 2016). The authors evaluated which traits were also considered by local farmers for selecting desirable individuals, providing important highlights on which traits could be useful in conservation and breeding programs of *Butia* palms. 

### Physicochemical properties and nutrition

Although we separated morphological and physicochemical characteristics, both provide important traits that may be phenotyped for conservation and breeding endeavors with jelly palms. 

*Butia* species have been frequently studied for their physicochemical profile and antioxidant capacity. Studies have shown that jelly palms are rich in phenolic compounds, carotenoids, anthocyanins, tocopherols, minerals, vitamins, amino acids, and fatty acids (Faria et al., 2008b; Pierezana et al., 2015; Kobelnik et al., 2016; Barbosa et al., 2021; Morais et al., 2022; Wagner et al., 2022). In *B. capitata*, the distinct stages of ripening and storage conditions influenced compounds such as acetic acid, (E)- and (Z)-hex-2-enal, methoxyphenyloxyme, (E)-β-ocimene, α-fenchene and octyl methyl ether (Aguiar et al., 2014). High lipid concentration, beta-carotene, vitamin C and E, phenolics and copper were found in the pulp (Barbosa *et al.*, 2021). High oil content and dietary fiber were also discovered (Faria *et al.*, 2008a).

In *B. odorata*, 86 phenolic compounds were identified, with hydroxycinnamic acids and flavonols being the most common. After quantification by liquid chromatography, 4-hydroxybenzoic acid and catechin were identified as prevalent (Ma et al., 2019). The main volatile compound responsible for the fruit aroma was described as ethyl hexanoate (Bernardi et al., 2014). Sinapic and ellagic acid, trans-reveratrol, naringenin and apigenin were reported for the first time (Boeing et al., 2020). The fruits of *B. odorata* showed a rich composition in fiber, vitamin C, total carotenoids, and total phenolic content (Wagner et al., 2022). 

In *B. catarinensis* and *B. eriospatha*, high values of soluble solids were found, compared to other native fruits. High fiber values were also found, especially of the insoluble type (Rockett et al., 2020a). In another study with the same species, the authors performed *in vitro* assays and found 18 phenolic compounds in *B. catarinensis* and 28 in *B. eriospatha*. The main groups of phenolic compounds were hydroxybenzoic acids and flavonoid oils (Rockett *et al.*, 2020b). For the species *B. eriospatha*, higher ascorbic acid content and high antioxidant capacity for the peroxyl radical were denoted (Denardin et al., 2015). High carotenoid content, compared to other fruits, total flavonoid and phenolic content were also observed (Egea and Pereira-Netto, 2019). *B. witecki* and *B. lallemantii* seeds showed the presence of 25% fatty acids, high content of the phenolic compounds ferulic acid, luteolin, quercetin-3-rutinoside, isoquercetin and isorhamnetin (Rodrigues et al., 2022). 

Other studies have evaluated the composition of the oil present in *B. capitata*, as well as the characterization of the lipid content and fatty acid profile (Pierezana et al., 2015; Kobelnik et al., 2016). Kobelnik *et al.* (2016) demonstrated a predominance of saturated fatty acids at about 80% through chromatographic profiling. Oleic, palmitic and linolenic acids were also predominant, as well as unsaturated fatty acids. The main ester components from the transesterification of jelly palm oil were lauric acid (42.2%), capric acid (15.9%) and caprylic acid (14.6%) methyl and ethyl esters (Pierezana *et al.*, 2015; Teixeira et al., 2022). Isolation of triterpene methyl ethers from epicuticular waxes demonstrated a large amount of ethers, including extensive alkanol and triterpene methyl ether fraction (García et al., 1995). Regarding phenolic composition, variability was found in fruits after comparing genotypes of *B. catarinensis, B. odorata, B. paraguayensis* and *B. yatay* (Hoffmann et al., 2017).

In a sensory evaluation of ripe fruit of *B. capitata*, yellow-orange, juicy, fibrous and soft appearance were denoted. The pulp of mature fruits showed carbohydrates, lipids, proteins, carotenoids and pronounced juice acidity. Pulp senescence was related to decreased acidity, increased soluble solids, reduced firmness, nutrient levels, and increased phenolic accumulation (Ventura et al., 2022). Caproic acid methyl esters have indicated a link with fruit aroma perception (Lopes et al., 2012). As a feedstock for the synthesis of fatty acid methyl esters, the seed oil from the fruit of *B. capitata* and *B. Yatay* has been shown to be a suitable feedstock for biofuel, in accordance with the requirements of Brazilian, American and European agencies, with typical characteristics for use with fossil fuels and application in diesel engines (Zanuttini et al., 2014; Vieira et al., 2016). 

Studies have demonstrated antioxidant, anti-inflammatory and antimicrobial activity in *Butia*. The antioxidant potential seems to be related to the high concentration of carotenoids, being zeaxanthin the majority compound (Pereira et al., 2013; Otero et al., 2020; Teixeira et al., 2022). In *B. catarinensis*, Cruz et al. (2017) obtained extracts with strong antioxidant performance and bactericidal inhibition, mainly for Gram - negative bacteria. The main compounds identified were cinnamic and caprylic acid. The antioxidant potential in *B. eriospatha* was tested using the *Caenorhabditis elegans* animal model. Tambara et al. (2020) observed that extracts of *Butia* palm were able to prolong nematode (*C. elegans*) life cycle by protecting and reversing hydrogen peroxide-induced oxidative damage. *B. capitata* species, on the other hand, demonstrated functionality as an inhibitor of colorectal cancer cells (HT-29) in an *in vitro* antitumor activity analysis (Lahlou et al., 2022). *B. odorata* also demonstrated antitumor activity, this time on cervical cancer cell lines (SiHa and C33a) (Boeing et al., 2020). In the study by Vinholes et al. (2017), the fruits of *B. odorata* were shown to be promising sources for alpha-glucosidase inhibition and antioxidants and could be used to control blood glucose in patients with type 2 Diabetes mellitus. 

Industrial processing of jelly palms fruits, with subsequent pasteurization and freezing proved to degrade carotenoids and vitamin C. The bagasse, on the other hand, showed relative richness in total phenols and carotene. In terms of industrialization, juice pasteurization seems inadequate in the nutritional aspect, whereas the extraction of carotenoids and phenolic compounds proved relevant (Jachna et al., 2016). In a similar study, bioactive compounds and antioxidant capacity of commercial frozen pulp and fruits *of B. capitata* were compared. A large amount of vitamin C, quercetin and kaempferol derivatives were found as the main flavonoids present in the fruits. On the other hand, the frozen commercial pulps showed lower contents of bioactive compounds and antioxidant capacity compared to the fresh fruit (Genovese et al., 2008). 

### Population genetics and cytogenetics

Up to date, few molecular genetic studies with *Butia* species have been published. In a cytogenetic approach, the chromosomal characteristics of *Butia* species were investigated by Corrêa et al. (2009). All species (*B. capitata, B. eriospatha, B. odorata, B. paraguayensis* and *B. yatay*) had the same number of chromosomes (2n = 32). The species also have the same karyotypic formula: 14 metacentric, 12 submetacentric and 6 acrocentric chromosomes.

Population genetic studies are available, but very limited to the end of the years 2000 and the 2010s, using RAPD, AFLP, ISSR and SSR markers. Using ISSR markers, Gaiero et al. (2011) investigated the variability among four species - *B. paraguayensis, B. lallemantii, B. yatay* and *B. eriospatha*, and found high variability within populations of *B. paraguayensis*, *B. lallemantii* and *B. yatay*, possibly due to gene flow, past hybridization or life history traits. 

Using RAPD markers, Nunes et al. (2008) detected considerable genetic variability among 22 genotypes of *B. odorata* (the name was updated from the original paper, considering the reclassification proposed by Noblick, 2010). Using AFLP markers, Buttow et al. (2010) performed a molecular analysis of variance in eight populations of *B. odorata* (species name updated) and found that 83.68% of the genetic variability was attributed to variation within populations and 13.67% to differences between populations within the regions investigated. The results suggest that the populations have a common origin and may have undergone selection, drift, geographic isolation and mutations that caused the differences between them, structuring them into subpopulations (Buttow *et al.*, 2010). 

Using 14 newly developed SSR markers, Nazareno, Reis and collaborators studied the variability in *B. eriospatha*. They validated the use of SSR as an important marker for studying population genetics and evolution (Nazareno and dos Reis, 2011; Nazareno *et al.*, 2011). In another study, combining SSR data with reproductive biology data, they concluded that *B. eriospatha* was predominantly outcrossing, with a certain degree of biparental inbreeding. Self-compatibility and geitonogamy seemed to be present in isolated populations (Nazareno and dos Reis, 2012). By using nine microsatellite loci in four populations of *B. eriospatha* from Southern Brazil, high levels of genetic differentiation were found. The amount of observed heterozygosity differed significantly between small and large populations, indicating that small populations are more susceptible to genetic drift (Nazareno and dos Reis, 2013). In a comparison of the genetic diversity between wild and urban *B. eriospatha* populations, authors found greater variation in the urban species. The expected heterozygosity within wild populations was lower (*H*_*E*_ = 0.48) than in an urban population (*H*_*E*_ = 0.62) (Nazareno and dos Reis, 2014).

The SSR markers developed by Nazareno et al. (2011) were also transferable to *B. catarinensis*, with 86% of the markers successfully amplified. Moreover, the results indicated that there is a high potential for transfer of SSR markers between species of the same genus in the Arecaceae family. As for genome sequencing, we identified only one paper that characterized the plastidial genome of *B. eriospatha*. The complete sequence was 154,048 bp in length, with the typical quadripartite structure, consistent with other six species from tribe Cocoseae (Magnabosco et al., 2020).

### *Butia* nucleotide sequences

After searching GenBank for sequences available, at least one sequence has been registered for *B. capitata*, *B. eriopatha*, *B. paraguayensis*, *B. yatay*, *B. marmorii*, *B. lallemantii* and *B. odorata* (registered as *B. capitata* var *odorata*). Moreover, a few sequences were registered as *B.* aff *yatay* and *B.* aff. *paraguayensis*, probably due to difficulties in determining their classification. We also found sequenced registered at the genus level only, as *Butia* sp. (Table 1). Of the 37 genes located, 22 were plastidial and 15 were nuclear. Most of the genes were useful for phylogenetic inferences and cladistic studies (e.g. *phytochrome B (PHYB); trnQ-rps16 intergenic spacer; trnD-trnT intergenic spacer; ribulose-1,5-bisphosphate carboxylase/oxygenase large subunit; NADH dehydrogenase subunit F (ndhF*)), and for population genetics and evolution (e.g. microsatellite *but10*). 

**Table 1 -:** GenBank sequences for *Butia* species (based on the search https://www.ncbi.nlm.nih.gov/popset/?term=butia) as of 29 April 2023. The accession numbers for all sequences are presented.

Gene	Species and accession numbers for sequences									
	*B. capitata*	*B. eriospatha*	*Butia sp.*	*B. paraguayensis*	*B. yatay*	*B. marmorii*	*B. lallemantii*	*B. capitata var. odorata*	*B. aff. yatay*	*B. aff. paraguayensis*
acetyl-CoA carboxylase beta subunit (accD) gene	MG437906.1									
ATP synthase beta subunit (atpB) gene	JX903942.1	AY044469.1								
chloroplast trnD gene, trnY and trnE genes and trnE-trnT intergenic spacer region		AY044516.1								
maturase K (matK) gene	MH551819.1									
	EU004870.1									
	JX903668.1									
microsatellite but10 sequence*		JF748782.1								
NADH dehydrogenase subunit F (ndhF) gene	EU004887.1	AY044565.1								
	MG647202.1									
	JX903522.1									
phosphoribulokinase-like protein (PRK) gene		JQ821972.1								
phosphoribulokinase-like protein 2 (PRK) gene	AY601252.1									
	AY601251.1	AY601254.1								
		AY601253.1								
photosystem II protein D1 (psbA) and psbA-trnH intergenic spacer	OL312423.1									
photosystem II protein D1 (psbA), psbA-trnH intergenic spacer and tRNA-His (trnH) gene	OK469471.1									
phytochrome B (PHYB) gene		JQ822073.1		MK102235.1						
ribosomal protein S16 (rps16) gene	MG647461.1									
ribulose-1,5-bisphosphate carboxylase/oxygenase large subunit (rbcL) gene	KY627008.1	AY044632.1	MK753383.1							
	MG437645.1	MK753958.1	MK753382.1							
	JX903252.1	MK753956.1	MK753381.1							
	MK753957.1		MK753380.1							
	MK753955.1		MK753379.1							
	MK753954.1		MK753378.1							
			MK753377.1							
			MK753376.1							
			MK753375.1							
RNA polymerase subunit C1 (rpoc1) gene	MG438159.1									
rps16 gene	EU004908.1									
serine/threonine protein kinase (CISP4) gene				MK102259.1						
serine/threonine protein kinase RLCKVII (CISP4) gene		JQ822034.1								
tRNA-Leu (trnL) gene, trnL-trnF intergenic spacer and tRNA-Phe (trnF) gene	EU004864.1									
trnD-trnT intergenic spacer				MK102099.1						
trnK gene and maturase K (matK) gene	MK704816.1	MK704817.1	MK704787.1							
	MK704814.1	MK704815.1	MK704784.1							
	MK704813.1		MK704783.1							
			MK704782.1							
			MK704781.1							
			MK704780.1							
			MK704779.1							
			MK704778.1							
			MK704777.1							
trnQ-rps16 intergenic spacer		EF605537.1								
trnQ(UUG)-rps16 intergenic spacer and ribosomal protein S16 gene		AY044612.1								
WRKY transcription factor 2 (WRKY2) gene	FJ956951.1	FJ956954.1		FJ956956.1	FJ956958.1	FJ956955.1	FJ956957.1	FJ956953.1	FJ956950.1	
								FJ956952.1		
WRKY transcription factor 6 (WRKY6) gene	FJ957096.1	FJ957099.1		FJ957102.1	FJ957103.1	FJ957101.1	FJ957100.1	FJ957098.1	FJ957095.1	FJ957094.1
								FJ957097.1		
WRKY transcription factor 7 (WRKY7) gene	FJ957170.1	FJ957173.1		FJ957176.1	FJ957177.1	FJ957175.1	FJ957174.1	FJ957172.1	FJ957169.1	FJ957168.1
								FJ957171.1		
WRKY transcription factor 12 (WRKY12) gene	FJ957242.1	FJ957245.1		FJ957248.1	FJ957249.1	FJ957247.1	FJ957246.1	FJ957244.1	FJ957241.1	FJ957240.1
WRKY transcription factor 16 (WRKY16) gene	FJ957310.1	FJ957313.1		FJ957316.1	FJ957317.1	FJ957315.1	FJ957314.1	FJ957312.1	FJ957309.1	FJ957308.1
								FJ957311.1		
										
WRKY transcription factor 19 (WRKY19) gene	FJ957381.1	FJ957384.1		FJ957387.1	FJ957388.1	FJ957386.1	FJ957385.1	FJ957383.1	FJ957380.1	FJ957379.1
								FJ957382.1		
WRKY transcription factor 21 (WRKY21) gene	FJ957023.1	FJ957026.1		FJ957028.1	FJ957029.1		FJ957027.1	FJ957025.1	FJ957022.1	FJ957021.1
								FJ957024.1		

*This is the reference sequence for the microsatellite. Other sequences with their polymorphisms are available from NCBI.

In general, phylogenetic studies were not devoted to clarifying differences specifically for genus *Butia*, but rather to comprehend the relationships among palms in general. Two of the most important molecular phylogenetic studies used *WRKY* genes (Table 1), transcription factors involved in abiotic stresses. The comparison of sequences of a few paralogs among *Butia* species indicate that the genus constitutes a monophyletic group (Meerow et al., 2009; Meerow *et al.*, 2015).

## Discussion

This review provided a panorama of the phenotypic and genetic diversity studied so far for jelly palms. As per the bibliometric analysis, limited to our categorization, most studies were dedicated to physicochemical properties and nutrition facts of *Butia* species. A wide phenotypic variation for *Butia* palms, as revealed from the characterization of fruits with very different biometric, physicochemical and sensory properties is available from the literature. The indication of important traits that should be considered in the characterization of jelly palms (Mistura et al., 2016), as well as the vulnerability of each species (Eslabão et al., 2016), provide foundations for establishing conservation and breeding programs for these unique species. 

With regards to species delimitation, the literature available is pretty much based on morphological traits, whether they can be screened from a naked eye or evaluated from ultrastructural profiles (anatomical analyses). By searching GBIF, the number of registered species is higher that what we found on Web of Science. The database provided records for 27 distinct species, that can be explained mainly by older publications and registered observations that can be found on the database, as well as hybrid taxa. In fact, there are substantial morphological differences among species, such as *B. odorata* and *B. capitata*. However, at the molecular level, a few phylogenetic markers provided nuanced differences among the proposed species, but not enough to actually prove them to be distinct at the nucleotide level. The debate on whether they are distinct species could go to the biological concept, stated by Mayr, that defines species as a group of potential interbreeding populations that are reproductively isolated from other groups. That stated, it is necessary to further explore whether the taxonomic delimitations based on morphological characters are consistent with molecular analyses. Moreover, there are reports of hybrids between *Butia* and *Syagrus* species (Engels et al., 2021; Silveira et al., 2022).

As for traits related to chemical compounds and nutritional facts, *Butia* species demonstrate to be great sources of bioactive compounds and antioxidants, with potential as functional food for the treatment of cancer cells and glycemic control in patients with diabetes (Vinholes et al., 2017; Boeing et al., 2020, Lahlou et al., 2022). In this regard, further studies are needed to investigate the antitumor potential in different cancer cells, expanding the evidence so far demonstrated in *in vitro* studies. 

The phenolic composition varied among the *Butia* species evaluated (Hoffmann et al., 2018; Rockett et al., 2020a; Rodrigues et al., 2022) but in all, fruits have proved to hold excellent functional, nutritional, antioxidant, antimicrobial and antitumor properties. The broad functional potential for the cosmetic, pharmaceutical, industrial and food industries demonstrated in the reviewed studies, makes *Butia* a certainly rich product and still little or poorly explored. 

The molecular genetic studies so far developed have proven to be scarce and with important gaps. Usually, studies are limited to a few populations. Broader analyses and using single nucleotide polymorphisms (SNP) should be the goal of novel endeavors toward unraveling more specific as well as a broader context of range of distribution of jelly palms, so that a detailed panorama of their genetic conservation status might be described. Whole genome sequences of *Butia* taxa also need to be obtained, considering that only a few short sequences are available for conserved phylogenetic markers, as revealed by our search on NCBI. 

Another line of studies that has been increasingly employed among plant species is epigenetics or epigenomics. Since epigenetics deals with changes in gene expression due to modifications of DNA that do not alter the nucleotide sequence, such as DNA methylation, histone acetylation and other mechanisms, the environment is an important component to affect cellular mechanisms beyond genetic determination. As per our review, jelly palms have a broad phenotypic variation and populations are usually separated by considerable distances and in distinct climates and soils. Therefore, we believe that not only genetic adaptive mechanisms, such as Candido-Ribeiro et al. (2019) reported, but also epigenetic(epigenomic) processes are adaptive among the distinct populations. 

The prominent morphological differences among jelly palms led taxonomists to distinguish more than 20 species to this date. In fact, the differences are remarkable when we compare *Butia* taxa. At the genomic level, we have little information available for conducting phylogenomic analyses to further determine the levels of differentiation among the taxa. Either ways or both ways together, we need rapid and increasing studies toward conservation of jelly palms. Population viability analyses should be conducted coupling genomic resources as well as an in-depth characterization of the phenotypic variation available, as the literature showed that individuals of several populations are frequently old and no regeneration is occurring. This will ultimately lead to much more damage that could be repaired, so urgent measures need to be pursued for conservation of the genetic resources of these important and unique habitats that butiás compose, the palm groves. 

## Supplementary material

The following online material is available for this article:

Table S1 - Journals, number of papers involving *Butia* species in the subjects addressed in this review and impact factors (2021) at the time they were searched.

Table S2 - List of papers selected: category, *Butia* species and main findings.
