# Supplementary material for: Phenotypic and molecular basis for genetic variation in jelly palms (Butia sp.): where are we now and where are we headed to?
Source: Genet Mol Biol. 2023 Nov 10;46(3 Suppl 1):e20230145. doi: 10.1590/1678-4685-GMB-2023-0145 (PMC10637346; doi:10.1590/1678-4685-GMB-2023-0145)
Supplement: Table S1 - [file 1415-4757-GMB-46-3-s1-e20230145-s1.pdf]

Genetics and Molecular Biology
https://doi.org/10.1590/1678-4685-GMB-2023-0145

Supplementary Material to "Phenotypic and molecular basis for genetic variation in jelly palms (*Butia* sp.): where are we now and where are we headed to?"

Table S1 - Journals, number of papers involving *Butia* species in the subjects addressed in this review and impact factors (2021) at the time they were searched.

Journal	Nº	Impact Factor
Revista Brasileira de Fruticultura	8	1,1
Food Research International	4	7,4
Food Bioscience	3	5,3
Food Analytical Methods	2	3,4
Ciência Rural	2	0,9
American Journal of Botany	2	3,3
Biota Neotropica	2	1,4
Journal of Heredity	2	2,6
Brazilian Journal of Biology	2	1,6
Food Science and Technology	2	2,6
Journal of Food Biochemistry	1	3,6
Conservation Genetics	1	3,0
Journal of Supercritical Fluids	1	4,5
Energy Conversion and Management	1	11,5
Acta Botânica Brasilica	1	1,3
Botany Letters	1	1,5
Journal of Food Processing and Preservation	1	2,6
Aob Plants	1	3,1
Journal of Thermal Analysis and Calorimetry	1	4,7
Food Chemistry	1	9,2
Semina-Ciências Agrárias	1	0,5
Ciência Florestal	1	0,6
Acta Oecologica - International Journal of Ecology	1	1,9
Nutrients	1	6,7
Journal of Food Composition and Analysis	1	4,5
Pakistan Journal of Botany	1	1,1
Antioxidants	1	7,6
Phytochemistry	1	4,0
Journal of the Science of Food and Agriculture	1	4,1
Phytotherapy Research	1	6,3
Molecules	1	4,9
Plant Systematics and Evolution	1	1,6
Revista Chilena de Nutricion	1	0,1

Journal	Nº	Impact Factor
Palynology	1	1,9
Food Science and Technology International	1	2,6
Phytokeys	1	1,3
Food Science and Technology Research	1	0,7
Plant Genetic Resources - Characterization and Utilization	1	1,2
Fruits	1	0,5
Allelopathy Journal	1	0,9
Química Nova	1	1,1
Fuel	1	8,0
Scientia Horticulturae	1	4,3
Genetics and Molecular Biology	1	2,0
Anais da Academia Brasileira de Ciências	1	1,8
Interciencia	1	0,3
European Food Research and Technology	1	3,4
Journal of Agricultural and Food Chemistry	1	5,8
Journal of Food and Drug Analysis	1	6,1
