# Supplementary material for: Phenotypic and molecular basis for genetic variation in jelly palms (Butia sp.): where are we now and where are we headed to?
Source: Genet Mol Biol. 2023 Nov 10;46(3 Suppl 1):e20230145. doi: 10.1590/1678-4685-GMB-2023-0145 (PMC10637346; doi:10.1590/1678-4685-GMB-2023-0145)
Supplement: Table S2 - [file 1415-4757-GMB-46-3-s1-e20230145-s2.pdf]

Genetics and Molecular Biology
https://doi.org/10.1590/1678-4685-GMB-2023-0145

Supplementary Material to "Phenotypic and molecular basis for genetic variation in jelly palms (*Butia* sp.): where are we now and where are we headed to?"

Table S2 – List of papers selected: category, *Butia* species and main findings.

Category	Authors	Journal	Butia Species	Findings
Physicochemical properties and nutrition	Aguiar *et al.* (2014)	Food Research International	*B. capitata*	Volatile compounds were influenced by stages of its maturation and storage times. Ethyl hexanoate is indicated to be involved with the aroma of fruits over their maturation.
	Barbosa *et al.* (2021)	Food Science and Technology	*B. capitata*	Pulp yield was considerably high (68.59%). Pulp has high lipid content and total energy. Pulp contains beta-carotene, vitamin E, and high concentrations of vitamin C, phenolics and copper. The pulp is a source of carotenoids, vitamin C, copper and total phenolics, both natural antioxidants.
	Barcia *et al.* (2010)	Semina Ciências Agrárias	*B. capitata*	Fruits are good sources of bioactive compounds. All fruits showed concentrations of L-dehydroascorbic acid higher than L-ascorbic acid.
	Bernardi *et al.* (2014)	Food Analytical Methods	*B. odorata*	The principal volatile compounds identified as responsible for the characteristic aroma of jelly palm are ethyl hexanoate (positively) and hexanoic acid (negatively).
	Boeing *et al.* (2020)	Food Analytical Methods	*B. odorata*	Sinapic and ellagic acids, trans-resveratrol, naringenin, and apigenin were reported in *B. odarata* fruits for the first time. *B. odorata* fruits showed antitumor activity against cervical cancer cell lines (SiHa and C33a).
	Cruz *et al.* (2017)	Journal of Supercritical Fluids	*B. catarinensis*	The extracts presented antioxidant performance and were considered strong bacterial inhibitors. The main compounds were cinnamic acid and caprylic acid. In general, extracts were more effective against Gram-negative bacteria.

Category	Authors	Journal	Butia Species	Findings
	Denardin *et al*. (2015)	Journal of Food and Drug Analysis	*B. eriospatha*	*Butia* had the highest content of ascorbic acid, although its antioxidant activity in the DPPH and FRAP assays were the lowest. In the TRAP assay, purple-fleshed pitanga, cherokee blackberry, and butiá showed the highest capacity to scavenge the peroxyl radical.
	do Vale *et al*. (2021)	Biota Neotropica	*B. capitata*	After analyzing yeast species in native trees from Cerrado, the fruits of *B. capitata* presented the highest species richness. *Candida* and *Meyerozyma* were the most frequent genera.
	Egea and Pereira-Netto (2019)	Europeand Food Research and Technology	*B. eriospatha*	Total carotenoid content was significantly higher in jelly palm fruits. Total flavonoid content was significantly higher.
	Faria *et al*. (2008a)	Revista Brasileira de Fruticultura	*B. capitata*	*Butia* pulp presented high content of oil, dietetic fiber, pro-vitamin, vitamin C, phenolic compounds and potassium. *Butia* shows high potential for food enrichment in a local community. Results showed the high value of products supplied by small farmers, presenting the cultural importance of *B. capitata*.
	Faria *et al*. (2008b)	Revista Brasileira de Fruticultura	*B. capitata*	*Butia* nut presented 9,9% of moisture, 57,8% of total lipid, 25,8% of neutral detergent fiber, 17,6% of acid detergent fiber and 1,6% of ash. The fat from *Butia* nut presented high content of lauric acid (42,1%), followed by oleic acid (16,9%). The saturated fatty acids predominated (78,9%), mainly the medium chain length fatty acids (58,3%).
	Faria *et al*. (2011)	Revista Brasileira de Fruticultura	*B. capitata*	Results suggested that *B. capitata* pulp may be a good source of β-carotene and provitamin A.
	Ferrão et al. (2017)	Food Science and Technology Research	*B. odorata*	The water activity of the dried pulp ranged from 0.43 to 0.51. The best results were obtained using MW at 60°C, because this presented no significant sensory differences and required a shorter drying time than MW at 50°C.
	García *et al*. (1995)	Phytochemistry	*B. capitata*	*B. capitata* contain large amounts of ethers. In *Butia* species, the alkanol plus triterpene methyl ether fractions represent 60-80%.
	Genovese *et al*. (2008)	Food Science and Technology International	*B. capitata*	*B. capitata* showed a significant vitamin C content. Quercetin and kaempferol derivatives were the main flavonoids present in all samples. All commercial frozen pulps presented lower contents of bioactive compounds and antioxidant capacity than their respective fruits.
	Hoffmann *et al*. (2017)	Journal of Agricultural and Food Chemistry	*B. catarinensis, B. odorata, B. paraguayensis* and *B. yatay*	Liquid chromatography–mass spectrometry based metabolic profiling coupled with chemometric analysis can be used to discriminate among *Butia* species and between geographical origins of *B. odorata* and to identify primary and specialized metabolites responsible for their discrimination.

Category	Authors	Journal	Butia Species	Findings
	Hoffmann *et al*. (2018)	Fruits	*B. catarinensis, B. odorata, B. paraguayensis and B. yatay*	The phenolic composition varied among four species evaluated and was dependent on the collection site for *B. odorata*. The abundant phenolics make butia fruit an excellent source of natural antioxidants for food and pharmaceutical applications.
	Jachna *et al*. (2016)	Journal of the Science of Food and Agriculture	*B. capitata*	The evaluation of *Butia* pomace showed that it is relatively rich in total phenols and in-carotene. From the nutrition viewpoint, pasteurized juice does not seem adequate. On the other hand, extraction of carotenoids and phenolic compounds from the pomace appears to be a relevant process.
	Kobelnik *et al*. (2016)	Journal of Thermal Analysis and Calorimetry	*B. capitata*	A chromatographic profile of *B. capitata* oil revealed a predominance of saturated fatty acids. The results of gas chromatographic analysis showed that this oil has a high amount of saturated fatty acids, which corresponds to around 80%.
	Lahlou *et al*. (2022)	Food Bioscience	*B. capitata*	*B. capitata* contains high amounts of medium-chain saturated fatty acids, a good source of lauric acid. The methanol:water extracts of fruits induced dose-and time-dependent inhibitory effects on HT-29 cancer cells. Overall, the fruits of Arecaceae taxa evaluated constitute suitable candidates to be used as functional foods.
	Lopes *et al*. (2012)	Revista Brasileira de Fruticultura	*B. capitata*	Oleic and palmitic acids predominated in *B. capitata* and other species; all presented prevalence of unsaturated fatty acids.
	Ma *et al*. (2019)	Antioxidants	*B. odorata*	This study indicated that *B. odorata* fruits are a good source of polyphenols and has strong antioxidant potential for health promotion.
	Magalhães *et al*. (2012)	Allelopathy Journal	*B. capitata*	Methanol extracts of endosperm and endocarp did not affect germination, germination speed, fresh and dry weights of radicle and hypocotyl, but decreased the hypocotyl and radicle lengths. The principal allelopathic substances identified were: esters methyl (Z)-octadec-9-enoate, methylhexadecanoate and lauric, myristic, oleic, palmitic and linoleic acid.
	Morais *et al*. (2022)	Nutrients	Na	Arecaceae palm tree fruits have high nutritional value and are rich in bioactive compounds. Fruits also have potential for uses in food, pharmaceutical, biotechnology, and cosmetic industries.
	Otero *et al*. (2020)	Revista Chilena de Nutrición	*B. capitata*	*Butia* had a high concentration of carotenoids. *Butia* and the pupunha were those that presented the highest content of carotenoids. Among the fruits analyzed, those with the highest potential antioxidants were guava, java plum and butia.

Category	Authors	Journal	Butia Species	Findings
	Paroul *et al.* (2009)	Brazilian Journal of Biology	*Butia* sp.	The comparison of the means for the total crude wax yields showed significant differences among *Butia* and *Syagrus* samples.
	Pereira *et al.* (2013)	Journal of Food Composition and Analysis	*B. capitata*	*Butia* palm fruit showed elevated carotenoid content and greater antioxidant capacity. By the DPPH (2,2-dipheny1-1-picrylhydrazyl) method, *Butia* palm fruit and mandacaru-de-tres-quinas fruit were considered to have the same antioxidant potential with no difference between them.
	Pierezana *et al.* (2015)	Quimica Nova	*B. capitata*	Lauric acid (42.2%), capric acid (15.9%) and caprylic acid (14.6%) methyl and ethyl esters were the main ester components of transesterification of the oil from *B. capitata*.
	Rockett et al. (2020a)	Food Research International	*B. catarinensis and B. eriospatha*	Phenolic compounds were identified in *B. catarinensis*, *B. eriospatha* and *Arumbeva*. The main groups of phenolic compounds found in the fruits were hydroxybenzoic acids, flavan-3-ols and flavonols. In hydroethanolic extracts of *B. catarinensis* and *Arumbeva*, the total phenolic content increased by around 67% and 35%, respectively.
	Rockett *et al.* (2020b)	Journal of Food Processing and Preservation	*B. catarinensis and B. eriospatha*	The value obtained for soluble solids of *B. catarinensis* was higher. *B. eriospatha* exhibited the highest and lowest values for magnesium. In general, the fruits evaluated presented high moisture content, low lipid content, and a good amount of fiber.
	Rockett *et al.* (2021)	Food Bioscience	*B. catarinensis*	The use of packing was able to increase softness, maintain carotenoid levels, the antioxidant capacity of the fruit, and suffer less significant ascorbic acid losses. The use of packages combined with cooling (5 degrees C) was sufficient to prolong the shelf life for up to 25 days.
	Rodrigues *et al.* (2022)	Food Research International	*B. catarinensis, B. lallemantii, B. odorata, B. witecki*	25% of the fatty acids in the seeds of *B. lallemantii*, *B. odorata*, *B. witeckii*, and *Syagrus romanzoffiana* were found to be unsaturated. A high content of the phenolic compounds ferulic acid, luteolin, quercetin-3-rutinoside, isoquercetin, and isorhamnetin were found in *B. odorata*, *B. catarinensis*, and *B. witeckii*.
	Schneider *et al.* (2017)	Phytotherapy Research	Na	*Butia* showed some antioxidant, anti-inflammatory, and antimicrobial activity. Clinical studies must be conducted to confirm the effectiveness of *Butia* sp.
	Tambara *et al.* (2020)	Journal of Food Biochemistry	*B. eriospatha*	*Butia* extract increased *C. elegans* lifespan under stress. The *Butia* is able to extend the lifespan of the nematode *C. elegans* and this effect may be mediated by an induced resistance to oxidative stress.

Category	Authors	Journal	Butia Species	Findings
	Teixeira *et al.* (2022)	Molecules	Na	Myristic, caprylic, capric, and lauric acids are the main saturated fatty acids, while oleic acid is the main unsaturated. Carotenoids and phenolic compounds are the main bioactive compounds, contributing to high oxidative stability.
	Ventura *et al.* (2022)	Scientia Horticulturae	*B. capitata*	The fruits had a yellow-orange, succulent, fibrous, and soft aspect, with reduced phenolic contents in the vacuoles and high levels of soluble solids. Pulp senescence is related to a decline in acidity, reduction in firmness, in nutrient levels, and increased phenolic accumulations.
	Vieira *et al.* (2016)	Fuel	*B. capitata*	*B. capitata* proved to be suitable starting material for biofuel according to the requirements of Brazilian, American and European agencies and has the typical characteristics for use with fossil fuel and the possibility of application in diesel-based engines without drastic performance changes.
	Vinholes *et al.* (2017)	Food Bioscience	*B. odorata*	All fruits were rich in total phenolic compounds; jelly palms were the richest ones in non-reducing sugars. Native fruits are promising sources of alpha-glucosidase inhibitors and antioxidants that can be used to control glycemia in patients with type 2 Diabetes mellitus.
	Zanuttini *et al.* (2014)	Energy Conversion and Management	*B. yatay*	*B. Yatay* coconut oil had acid values between 109 and 140 mg KOH/g, and phosphorus content in the order of 600 ppm. The kinetic constant for the esterification reaction rapidly decreased as a function of time, due to the consumption of the catalyst by the alkyl-sulphate formation reaction.
	Wagner *et al.* (2022)	Food Science and Technology	*B. odorata*	Fruits showed a rich composition in fibers, vitamin C, total carotenoids, and total phenolic content, which contribute to health maintenance. *Butia* fruits represent a potential product for nutritional enrichment in diets.
Plant morphology	Bobrov and Romanov (2019)	Botany Letters	*B. capitata*	Twenty-seven morphogenetic fruit types are recognized in the research and their probable modes of transformations are described based on original data and earlier studies.
	Candido-Ribeiro *et al.* (2019)	Acta Oecologica - International Journal of Ecology	*B. eriospatha*	Greater variation was observed within the forest population. The grassland population showed a greater proportion of pulp per fruit, but smaller seeds, which may suggest plasticity, local adaptation, or both. The average production of infructescence per individual is lower in the forest environment.
	da Silva and Scariot. (2013)	Acta Botanica Brasilica	*B. capitata*	Fruit biometric variables differ between the populations of *B. capitata* in Cerrado. The productivity of fruits also differs between study sites sampled and is related to the height of individuals and to their foliar biomass.
	de Moura *et al.* (2010)	Biota Neotropica	*B. capitata*	The fruit pulp represents approximately 80% of the fruit. The mass and diameter of the fruit showed significant and positive correlations, suggesting that fruits of bigger size and mass have heavier pulp and pyrene and more seeds per fruit.
	Guilherme *et al.* (2015)	Brazilian Journal of Biology	*B. purpurascens*	The harvested sites produced significantly fewer leaves, spathes, inflorescences and infructescence than the non-harvested sites. The supply of resources to the local fauna is possibly reduced in sites

Category	Authors	Journal	Butia Species	Findings
				under leaf exploitation, which in the long term can represent damage to the palm tree population structure and dynamics.
	Mistura *et al*. (2016)	Plant Genetic Resources	*B. odorata*	Research developed a list of descriptors for *B. odorata*. Interactions with farmers led to the identification of only five descriptors: fruit size, number of bunches per plant, presence of fibers in the pulp, fruit flavor and color of mature fruit.
	Mourelle *et al*. (2016)	Palynology	*B. eriospatha*, *B. odorata*, *B. paraguayensis*, *B. yatay*	Results showed that pollen viability of all species of *Butia* analyzed was high enough to ensure good pollination. Therefore, pollen viability is not the limiting factor for population continuity.
	Noblick and Santanna-Santos (2021)	Phytokeys	*B. eriospatha*, *B. odorata*, *B. paraguayensis*, *B. yatay*	The importance of a broader sampling exercise when studying leaf anatomy, due to possible ecological and developmental variations that may occur in some species, was emphasized.
	Rocha *et al*. (2022)	Ciência Rural	*B. purpurascens*	Yellow morph showed larger and heavier fruits than magenta morph. Morphometric differences were also evident among the populations, suggesting that ecosystems fragmentation can cause deleterious genetic effects in *B. purpurascens* in long-term.
	Sant'anna-Santos *et al*. (2015)	Anais da Academia Brasileira de Ciencias	*B. capitata and B. odorata*	Leaf anatomy showed exclusive characters for *B. marmorii* and *B. matogrossensis*, reliable anatomical characters, especially the raphides, were valuable in species distinction.
	Sant'anna-Santos *et al*. (2018)	AOB Plants	*B. archeri*, *B. campicola*, *B. capitata*, *B. catarinensis*, *B. eriospatha*, *B. exospadix*, *B. lallemantii*, *B. leiospatha*, *B. leptospatha*, *B. lepidotispatha*, *B. marmorii*, *B. matogrossensis*, *B. microspadix*, *B. paraguayensis*, *B. pubispatha*, *B. purpurascens e B. yatay*.	Anatomical keys presented relevant characters that allow the identification of the recognized species of *Butia*. Reliable anatomical characters of easy observation, especially the raphides, are valuable in species distinction.

Category	Authors	Journal	Butia Species	Findings
	Schlindwein *et al*. (2017)	Ciência Rural	*B. odorata*	Data analysis revealed a strong correlation between the edaphic conditions and the yield from the *Butia* palms. Tapes *Butia* palms exhibited higher fruit yield. The lowest fruit yields were linked to nutrient-poor soils in Brachiaria pastures, as well as sandy plains containing high levels of sodium.
	Soares and Longhi (2011)	Ciência Florestal	*B. witeckii*	*B. witeckii* was close to *B. paraguayensis* e *B. yatay*, differing from these two species by the size and weight of the fruit, size, weight and shape of the endocarp/pyrene, and by the number of pinnae (leaflets) on each side of rachis.
Plant morphology, Physiochemistry and nutrition	Beskow *et al*. (2015)	Food Chemistry	*B. odorata*	Genotype 117 was the highest yielding, with an estimated fruit yield of 22,000 kg ha and pulp yield of 12,000 kg ha. None of the genotypes evaluated showed high levels of fruit yield and bioactive phytochemical content.
	Ferrão *et al*. (2013)	Food Research International	*B. odorata*	It was possible to discriminated samples from different regions mainly due to different total lipid content, fatty acids profile and color parameters.
	Nunes *et al*. (2010)	Interciencia	*B. capitata*	It was possible to differentiate butia palm genotypes in relation to size, weight, number of fruits, firmness, color, acidity, and total soluble solids in a population of 121 plants.
	Schwartz *et al*. (2010)	Revista Brasileira de Fruticultura	*B. capitata*	Properties and/or genetic variations among the populations of *B. capitata* provided variability for the duration of the cycle, color of the epidermis of the fruits, volume of juice produced, relation between total soluble solids and titratable acidity, biometric characteristics of fruit and annual productivity.
Population genetics and cytogenetics	Buttow *et al*. (2010)	Revista Brasileira de Fruticultura	*B. capitata*	83.68% of the genetic variability is attributed to variation within populations and 13.67% attributed to differences between populations within regions. There is presence of genetic variability among all populations, without subdivision due to geographic isolation.
	Corrêa *et al*. (2009)	Revista Brasileira de Fruticultura	*B. capitata, B. eriospatha, B. odorata, B. paraguayensis and B. yatay*	All species studied had the same chromosome number, $2n = 2x = 32$, also having the same karyotypic formula. The karyotypes of all species are symmetrical, showing two pairs of satellite chromosomes, a pair of satellite metacentric chromosomes and a pair of satellite acrocentric chromosomes.
	Gaiero *et al*. (2011)	Plant Systematics and Evolution	*B. lallemantii, B. paraguayensis and B. yatay*	Genetic distance analyses indicate the existence of low variability among *Butia* species. Variability within populations was high, possibly due to gene flow, past hybridisation or life history traits.
	Magnabosco *et al*. (2020)	Genetics and Molecular Biology	*B. eriospatha*	The complete plastome sequence of B. eriospatha is 154,048 bp in length, with the typical quadripartite structure. This plastome encodes 113 unique genes, being 79 protein-code genes, 30 tRNA genes and four rRNA genes.
	Nazareno and dos Reis (2011)	American Journal of Botany	*B. eriospatha*	Study highlights that microsatellite molecular marker class can be a useful tool for population genetics and evolutionary studies for many plant species.

Category	Authors	Journal	Butia Species	Findings
	Nazareno and dos Reis (2012)	Journal of Heredity	*B. eriospatha*	*B. eriospatha* is a predominantly outcrossing species and certain degree of biparental inbreeding does occur. The species is self-compatible, and reproduction may also occur by geitonogamy. The effective population size was lower than that expected for panmictic populations.
	Nazareno and dos Reis (2013)	Journal of Heredity	*B. eriospatha*	Populations of *B. eriospatha* showed high levels of genetic differentiation. Populations investigated would be at an extremely high risk of local extinction, with a greater than 50% reduction in the effective population size, in the next 40 years.
	Nazareno and dos Reis (2014)	Conservation Genetics	*B. eriospatha*	The illegally traded *B. eriospatha* individuals had more genetic variation than all of the studied wild *B. eriospatha* populations. Urban *B. eriospatha* individuals came from a variety of different populations, with 46 % coming from other populations.
	Nazareno *et al*. (2011)	American Journal of Botany	*B. eriospatha*	New microsatellite markers were developed and described for *B. eriospatha*, and they have been shown to be applicable for other species from the *Butia* genus.
	Nunes *et al*. (2008)	Revista Brasileira de Fruticultura	*B. capitata*	A total of 136 fragments were obtained, 77 of which were polymorphic. With RAPD markers, it was possible to obtain a unique molecular profile and estimate of the existing variability between the evaluated genotypes.

References

Aguiar MCS, Silvério FO, Gevany PDP, Lopes PSN, Fidêncio PH and Ventura JS (2014) Volatile compounds from fruits of *Butia capitata* at different stages of maturity and storage. Food Res Int 62:1095-1099.

Barbosa MCA, Rosa QDS, Cardoso LDM, Gomides AFDF, Barbosa LCDA, Sant'anna HMP, Pinheiro SS, Peluzio MCG, Teixeira RDBL and Valente MAS (2021) Composition proximate, bioactive compounds and antioxidant capacity of *Butia capitata*. Food Sci Technol 41:763–768.

Barcia M, Jacques A, Pertuzatti P and Zambiazi R (2010) Determination by HPLC of ascorbic acid and tocopherols in fruits. Semin Cienc Agrar 31:381-389.

Bernardi G, Vendruscolo RG, Ferrão TDS, Barin JS, Cichoski AJ and Wagner R (2014) Jelly Palm (*Butia odorata*) Wine: Characterization of volatile compounds responsible for aroma. Food Anal Methods 7:1982–1991.

Beskow GT, Hoffmann JF, Teixeira AM, Fachinello JC, Chaves FC and Rombaldi CV (2015) Bioactive and yield potential of jelly palms (*Butia odorata* Barb. Rodr.). Food Chem 172:699-704.

Bobrov AVFC and Romanov MS (2019) Morphogenesis of fruits and types of fruit of angiosperms. Bot Lett 166:366-399.

Boeing J, Barizão E, Rotta E, Volpato H, Nakamura C, Maldaner L and Visentainer J (2020) Phenolic compounds from Butia odorata (Barb. Rodr.) noblick fruit and its antioxidant and antitumor activities. Food Anal Methods 13:61-68.

Buttow MV, Castro CM, Schwartz E, Tonietto A and Barbieri RL (2010) Caracterização molecular de populações de *Butia capitata* (Arecaceae) do sul do Brasil através de marcadores AFLP. Rev Bras Frutic 32:230–239.

Candido-Ribeiro R, Lauterjung MB, Montagna T, Bernardi AP, Freitas da Costa NC, Hoeltgebaum MP and dos Reis MS (2019) Distinct seeds in contrasting habitats: Morphological and reproductive responses in *Butia eriospatha* to new environmental conditions. Acta Oecol 99:103447.

Corrêa LB, Barbieri RL, Rossato M, Büttow MV and Heiden G (2009) Karyological characterization of *Butia* (Arecaceae) palm trees. Rev Bras Frutic 31:1111-1116.

Cruz PN, Pereira TCS, Guindani C, Oliveira DA, Rossi MJ and Ferreira SRS (2017) Antioxidant and antibacterial potential of butia *(Butia catarinensis*) seed extracts obtained by supercritical fluid extraction. J Supercrit Fluids 119:229-237.

da Silva PAD and Scariot A (2013) Phenology, biometric parameters and productivity of fruits of the palm *Butia capitata* (Mart.) Beccari in the Brazilian cerrado in the north of the state of Minas Gerais. Acta Bot Bras 27:580–589.

de Moura RC, Lopes PSN, Brandão Junior D da S, Gomes JG and Pereira MB (2010) Biometria de frutos e sementes de *Butia capitata* (Mart.) Beccari (Arecaceae), em vegetação natural no Norte de Minas Gerais, Brasil. Biota Neotrop 2:415–9.

Denardin CC, Hirsch GE, da Rocha RF, Vizzotto M, Henriques AT, Moreira JCF, Guma FTCR and Emanuelli T (2015) Antioxidant capacity and bioactive compounds of four Brazilian native fruits. J Food Drug Anal Sep 23:387-398.

do Vale HMM, dos Reis JBA, de Oliveira M, Moreira GAM and Bomfim CA (2021) Yeasts in native fruits from Brazilian neotropical savannah: Occurrence, diversity and enzymatic potential. Biota Neotrop 21:e20201184.

Egea MB and Pereira-Netto AB (2019) Bioactive compound-rich, virtually unknown, edible fruits from the Atlantic Rainforest: Changes in antioxidant activity and related bioactive compounds during ripening. Eur Food Res Technol 245:1081–1093.

Faria JP, Almeida F, Silva LCR da, Vieira RF and Agostini-Costa T da S (2008a) Caracterização da polpa do coquinho-azedo (*Butia capitata* var capitata). Rev Bras Frutic 30:827–9.

Faria JP, Arellano DB, Grimaldi R, Silva LCRD, Vieira RF, Silva DBD and Agostini-Costa TDS (2008b) Caracterização química da amêndoa de coquinho-azedo (*Butia capitata* var *capitata*). Rev Bras Frutic 30:549–552.

Faria JP, Siqueira EMA, Vieira RF and Agostini-Costa TS (2011) Fruits of Butia capitata (Mart.) Becc as good sources of β-caroteneand provitamina. Rev Bras Frutic 33:612–617.

Ferrão TS, Ferreira DF, Flores DW, Bernardi G, Link D, Barin JS and Wagner R (2013) Evaluation of composition and quality parameters of jelly palm (*Butia odorata*) fruits from different regions of Southern Brazil. Food Res Int 54:57-62.

Ferrão TS, Tischer B, Menezes MFSC, Hecktheuer LHR, Menezes CR, Barin JS, Michels L and Wagner R (2017) Effect of microwave and hot air drying on the physicochemical characteristics and quality of jelly palm pulp. Food Sci Technol 23:835-843.

Gaiero P, Mazzella C and Agostini G, Bertolazzi S and Rossato M (2011) Genetic diversity among endangered Uruguayan populations of *Butia* Becc. species based on ISSR. Plant Syst Evol 292:105–116.

García S, Heinzen H, Hubbuch C, Martínez R, de Vries X and Moyna P (1995) Triterpene methyl ethers from palmae epicuticular waxes. Phytochem 39:1381-1382.

Genovese MI, Da Silva MP, De Souza AESG and Lajolo FM (2008) Bioactive compounds and antioxidant capacity of exotic fruits and commercial frozen pulps from Brazil. Food Sci Technol Int 14:207-214.

Guilherme F, Vasconcelos E, Coelho C, Ressel K, Batista N and Souza L (2015) Vegetative and reproductive phenology of *Butia purpurascens* Glassman (Arecaceae) under the effects of leaf harvesting. Braz J Biol 75:77–85.

Hoffmann J, Crizel R, Madruga N, Barbieri RL, Rombaldi CV and Chaves F (2018) Flavan-3-ol, flavanone, flavone, flavonol, phenolic acid, and stilbene contents of four *Butia* species (Arecaceae). Fruits 73:125-137.

Hoffmann JF, Zandoná GP, dos Santos PS, Dallmann CM, Madruga FB, Rombaldi CV and Chaves FC (2017) Stability of bioactive compounds in butiá (*Butia odorata*) fruit pulp and nectar. Food Chem 237:638–644.

Jachna TJ, Hermes VS, Flôres SH and Rios AO (2016) Bioactive compounds in pindo palm (*Butia capitata*) juice and in pomace resulting of the extraction process. J Sci Food Agric 96:1216-22.

Kobelnik M, Fontanari GG, Marques MR, Ribeiro CA and Crespi MS (2016) Thermal behavior and chromatographic characterization of oil extracted from the nut of the *Butia* (*Butia capitata*). J Therm Anal Calorim 123:2517–2522.

Lahlou A, Chileh-Chelh T, Lyashenko S, Rincón-Cervera MA, Rodríguez-García I, López-Ruiz R, Urrestarazu M and Guil-Guerrero JL (2022) Arecaceae fruits: Fatty acids, phenolic compounds and in vitro antitumor activity. Food Biosci 50:102181.

Lopes RM, da Silva JP, Vieira RF, Silva da DB, Gomes IS and Agostini-Costa TDS (2012) Composição de ácidos graxos em polpa de frutas nativas do cerrado. Rev Bras Frutic 34:635–640.

Ma C, Dunshea FR and Suleria HAR (2019) LC-ESI-QTOF/MS characterization of phenolic compounds in palm fruits (Jelly and Fishtail Palm) and their potential antioxidant activities. Antioxidants 14:483.

Magalhães HM, Lopes PSN, Silvério FO and Silva HFJ (2012) Effects of *Butia Capitata* pyrenes extracts on the germination of lettuce seeds. Allelopathy J 30:49-60.

Magnabosco JWDS, Fraga HPDF, da Silva RS, Rogalski M, de Souza EM, Guerra MP and Vieira LN (2020) Characterization of the complete plastid genome of *Butia eriospatha* (Arecaceae). Genet Mol Biol 43:e20200023.

Mistura CC, Barbieri RL, Castro CM, Padulosi S and Alercia A (2016) Descriptors for on-farm conservation and use of *Butia odorata* natural populations. Plant Genet Resour 14:35-40.

Morais RA, Teixeira GL, Ferreira SRS, Cifuentes A and Block JM (2022) Nutritional composition and bioactive compounds of native Brazilian fruits of the arecaceae family and its potential applications for health promotion. Nutrients 14:4009.

Mourelle D, Gaiero P, Speroni G, Millán C, Gutiérrez L and Mazzella C (2016) Comparative pollen morphology and viability among endangered species of *Butia* (Arecaceae) and its implications for species delimitation and conservation. Palynology 40:160-171.

Nazareno AG and dos Reis MS (2011) The same but different: Monomorphic microsatellite markers as a new tool for genetic analysis. Am J Bot 98:e265-7.

Nazareno AG, Zucchi MI and dos Reis MS (2011) Microsatellite markers for *Butia eriospatha* (Arecaceae), a vulnerable palm species from the Atlantic Rainforest of Brazil. Am J Bot 98:e198-200.

Nazareno AG and dos Reis MS (2012) Linking phenology to mating system: Exploring the reproductive biology of the threatened palm species *Butia eriospatha*. J Hered 3:842–852.

Nazareno AG and dos Reis MS (2013) At risk of population decline? An ecological and genetic approach to the threatened palm species *Butia eriospatha* (Arecaceae) of Southern Brazil. J Hered 105:120-9.

Nazareno AG and dos Reis MS (2014) Where did they come from? Genetic diversity and forensic investigation of the threatened palm species *Butia eriospatha*. Conserv Genet 15:441–452.

Noblick LR and Santanna-Santos BF (2021) Diversity of leaf anatomy within a single leaflet and between leaflets of four *Butia* (Arecaceae, Arecoideae) species. PhytoKeys 180:31-52.

Nunes AM, Bianchi VJ, Fachinello JC, Carvalho AZ and Cardoso G (2008) Caracterização molecular de butiazeiro por marcadores RAPD. Rev Bras Frutic 30:702-707.

Nunes A, Fachinello J, Radmann E, Bianchi J and Schwartz E (2010) Morphological and physico-chemical characteristics of the jelly palm tree (*Butia capitata*) in the Pelotas Region, Brazil. Interciencia 35:500-505.

Otero D, Antunes B, Bohmer B, Jansen C, Crizel M, Lorini A, Krumreich F and Zambiazi RC (2020) Bioactive compounds in fruits from different regions of Brazil. Rev Chil Nutr 47:31-40.

Paroul N, Cansian R, Rossato M, Pauletti G, Serafini L, Rota L, Moyna P and Heinzen H (2009) Use of palmae wax hydrocarbon fractions as chemotaxonomical markers in *Butia* and *Syagrus*. Braz J Biol 69:353-361.

Pereira MC, Steffens RS, Jablonski A, Hertz PF, Rios A de O, Vizzotto M and Flôres SH (2013) Characterization, bioactive compounds and antioxidant potential of three Brazilian fruits. J Food Compos Anal 29:19-24.

Pierezana L, Cabral MRP, Martins ND, Stropa JM, Oliveira LCSD, Scharf DR, Simionatto EL, da Silva RCL and Simionatto E (2015) Composição química e temperatura de cristalização de ésteres obtidos de quatro óleos vegetais extraídos de sementes de plantas do Cerrado. Quím Nova 38:328–32.

Rocha JDL, Guilherme FAG, Rocha DI, Pereira K de AR, Coelho CP and de Souza LF (2022) Morphometry of fruits and pyrenes in two morphotypes and populations of *Butia purpurascens* Glassman (Arecaceae). Cienc Rural 52:e20210303.

Rockett F, Schmidt H, Rodrigues E, Flôres S and Rios A (2021) Application of refrigeration and packing can extend Butiá fruit shelf life. Food Biosci 42:101162.

Rockett FC, Schmidt HO, Pagno CH, Fochezatto ÉS, de Oliveira VR, da Silva VL, Flôres SH and Rios AO (2020a) Native fruits from southern Brazil: Physico-chemical characterization, centesimal composition, and mineral content. J Food Process Preserv.44:e14582.

Rockett FC, de Oliveira HS, Schmidt L, Rodrigues E, Tischer B, de Oliveira VR, da Silva VL, Rossini PA, Hickmann SF and Rios A (2020b) Phenolic compounds and antioxidant activity in vitro and in vivo of *Butia* and *Opuntia* fruits. Food Res Int 137:109740.

Rodrigues CE, Schäfer L, Gregolon JGN, de Oliveira JF, Perdomo Baez OP, Deolindo CTP, de Melo APZ, Singer RB, Kist TBL and Hoff R (2022) Determination of amino acid content, fatty acid profiles, and phenolic compounds in non-conventional edible fruits of seven species of palm trees (Arecaceae) native to the southern half of South America. Food Res Int 162:111995.

Sant'anna-Santos BF, Carvalho Junior WGO and Amaral VB (2015) *Butia capitata* (Mart.) Becc. lamina anatomy as a tool for taxonomic distinction from *B. odorata* (Barb. Rodr.) Noblick comb. nov (Arecaceae). An Acad Bras Cienc 87:71–81.

Sant'anna-Santos BF, dos Santos SA, Nunes ELP, Francino DMT and Carvalho Júnior WGO (2018) Does leaf anatomy aid in species identification of *Butia* (Arecaceae)? AoB Plants 28:ply046.

Schlindwein G, Tonietto A, Abichequer AD, de Azambuja AC, Lisboa BB and Vargas LK (2017) Pindo Palm fruit yield and its relationship with edaphic factors in natural populations in Rio Grande do Sul. Cienc Rural 47: e20151371.

Schneider LR, dos Santos DC, Campos AD and Lund RG (2017) The phytochemistry and pharmacology of *Butia* sp.: A systematic review and an overview of the technological monitoring process. Phytother Res 31:1495-1503.

Schwartz E, Fachinello J, Barbieri R and Silva J (2010) Performance of populations of *Butia capitata* of Santa Vitória do Palmar. Rev Bras Frutic 32:736-745.

Soares KP and Longhi JS (2011) Uma nova espécie de *Butia* (Becc.) Becc. (Arecaceae) para o Rio Grande do Sul, Brasil. Cienc Florest 21:203–208.

Tambara AL, da Silveira ÉC, Soares ATG, Salgueiro WG, Rodrigues CF, Boldori JR, de Ávila DS and Denardin CC (2020) Butiá fruit extract (*Butia eriospatha*) protects against oxidative damage and increases lifespan on *Caenorhabditis elegans*. J Food Biochem 44:e13139.

Teixeira GL, Ibañez E and Block JM (2022) Emerging lipids from Arecaceae palm fruits in Brazil. Molecules 29:4188.

Ventura LDM, Pereira GST, dos Santos HCM, de Lima JP, Simões MOM, Lopes PSN and Ribeiro LM (2022) Cytological aspects of *Butia capitata* (Arecaceae) fruit maturation and senescence. Sci Hortic 297:110938.

Vieira BM, Elicker C, Nunes CFP, Bairros AV, Becker EM, de Oliveira DM, Piva E, Fontoura LAM and Pereira CMP (2016) The synthesis and characterization of *Butia capitata* seed oil as a FAME feedstock. Fuel 184:533-535.

Vinholes J, Lemos G, Barbieri RL, Franzon RC and Vizzotto M (2017) *In vitro* assessment of the antihyperglycemic and antioxidant properties of araçá, butiá and pitanga. Food Biosci 19:92-100.

Zanuttini MS, Pisarello ML and Querini CA (2014) *Butia Yatay* coconut oil: Process development for biodiesel production and kinetics of esterification with ethanol. Energy Convers Manag 85:407-416.

Wagner JG, Cruz JG, Silveira T, Ferri NML, Richter VB, Lima FM, Figueira KU, Mistura CC, Vizzotto M and Barbieri RL (2022) Accessing the nutritional variability of *Butia odorata*: A food with identity. Food Sci Technol 42:e54822.
